# The Quality of Life and Psychosocial Implications of Cancer-Related Lower-Extremity Lymphedema: A Systematic Review of the Literature

**DOI:** 10.3390/jcm9103200

**Published:** 2020-10-02

**Authors:** Catharine Bowman, Katherine-Ann Piedalue, Mohamad Baydoun, Linda E. Carlson

**Affiliations:** Division of Psychosocial Oncology, Department of Oncology, Cumming School of Medicine, University of Calgary, Calgary, AB T2S 3C1, Canada; catharine.bowman@ucalgary.ca (C.B.); klpiedal@ucalgary.ca (K.-A.P.); mohamad.baydoun@ucalgary.ca (M.B.)

**Keywords:** lymphedema, cancer survivorship, psychosocial oncology, quality of life, psychosocial well-being, systematic review, health care delivery

## Abstract

Lower-extremity lymphedema (LEL) is a progressive, lifelong complication of cancer that places a substantial burden upon cancer survivors’ quality of life (QOL) and psychosocial well-being. Despite its prevalence, cancer-related LEL is inconsistently diagnosed, treated, and poorly recognized by health care professionals. The purpose of this systematic review was to summarize and appraise the quantitative literature evaluating the impact of cancer-related LEL on patients’ psychosocial well-being and QOL. Three databases (PubMed, PROQuest, and Scopus) were searched for observational research articles published before May 1st, 2020. Twenty-one articles were eligible (cross-sectional (*n* = 16), prospective cohort designs (*n* = 3), and retrospective cohort designs (*n* = 2)). The majority of studies reported a negative relationship between cancer-related LEL and global QOL and/or one or more psychosocial domains including (1) physical and functional; (2) psycho-emotional; (3) social, relational and financial. A greater number of LEL symptoms and higher LEL severity were associated with poorer QOL. Although the evidence to date suggests a negative relationship between cancer-related LEL and patients’ QOL and psychosocial well-being, there is a substantial need for longitudinal analyses to examine the directionality and temporality of this effect in order to inform cancer survivorship care modelling and improve patient outcomes after cancer.

## 1. Introduction

Lymphedema is a chronic, progressive and potentially disabling condition associated with cancer treatment and/or tumour obstruction of the lymphatic system [1]. Across all cancer types, upwards of 60% of people who have undergone surgery, radiation, and/or chemotherapy may later develop lymphedema after active treatment has subsided [2]. Furthermore, tumour obstructions of lymphatic vasculature or nodal regions can induce cancer-related secondary lymphedema development.

The literature has demonstrated that heightened psychological distress, depression, and anxiety are associated with the development of lymphedema after cancer [3,4]. These psychological sequelae are often linked to feelings of hopelessness, fear of the future and isolation due to immobility, social avoidance, and/or sexual dysfunction [4,5,6]. As a result of the skin changes and swelling associated with lymphedema, cancer survivors have also reported the negative impact of this condition on their appearance [3]. Considering the psychosocial complexity of life after cancer alone, these patients are now faced with a unique burden in the form of a secondary chronic illness that is associated with potential negative consequences on quality of life (QOL). QOL is a multifaceted concept that encompasses different domains of one’s life, including physical well-being, social/familial well-being, emotional well-being, and functional well-being [7]. Health-related QOL (HRQOL) specifically explores the physical, psychological, and social domains of health within the context of a person’s subjective experience [8], whereas global QOL is usually not referring specifically to the effects of a disease or condition on functioning. However, these terms are often used interchangeably and definitions of HRQOL and QOL vary within the literature [9]. Therefore, for the purposes of this systematic review, the terms HRQOL and global QOL are used as specified within each article reviewed.

### Cancer-Related Lower-Extremity Lymphedema

The cancer-related lymphedema literature primarily focuses upon breast cancer-related arm lymphedema, given that approximately 40% of breast cancer patients develop lymphedema after cancer treatment [10]. It is important to recognize, however, that lymphedema not only affects the upper extremities, but also lower extremities, trunk, and/or mixed regions of the body. Amongst other tumour groups, 53% of melanoma, 73% of gynecological, and 29% of prostate/penile cancer patients may develop lymphedema after cancer treatment [10,11,12]. Despite its localization, cancer-related lymphedema is classified under one individual disease, and yet it often poses unique challenges to patients depending upon its location.

Cancer-related lower-extremity lymphedema (LEL) symptoms include chronic swelling, regional heaviness, and pain [13]. Patients with LEL are at an increased risk for cancer recurrence and recurrent infections [13,14]. Advanced LEL may also evoke immobility, irreversible fibrosis, ulcers/skin breakages, and weeping of the lymphedematous region [4,15].

Similarly to upper-extremity lymphedema, patients experiencing cancer-related LEL are often required to comply with tedious and physically-demanding therapies to manage symptoms and prevent disease progression [16]. However, LEL often requires increased attentiveness, accommodation, and radical adjustments to activities of daily living (ADLs) in order to comply with treatment [17]. Constant disruptions to daily living and stressors associated with cancer-related LEL are not without financial repercussions. Cancer-related LEL costs have been reported as high as $2000 per month, with the majority of these costs not covered by public or private health plans [17,18]. The financial burden of cancer-related LEL may be further exacerbated due to patients’ inability to work following lymphedema onset [5].

Taken together, patients with cancer-related LEL may experience poor QOL and psychosocial sequelae due to numerous and complex challenges that include, but are not limited to decreased self-confidence, changes to social/familial relationships, psychological distress, loss of identity, emotional and financial stressors, and isolation as a result of their under-recognized and complex complication of cancer treatment [15,19]. Nevertheless, the majority of the relevant literature continues to primarily focus on upper-extremity breast cancer-related lymphedema. While research on upper-extremity cancer-related lymphedema should continue to expand, exploring and recognizing the unique deleterious effects of cancer-related LEL on patient QOL and psychosocial well-being is another critical prospect for future research. It is crucial to critically evaluate the current state of knowledge regarding the unique psychosocial burdens experienced by patients with cancer-related LEL in order to inform timely intervention and the development of novel care models of cancer survivorship to improve patient outcomes after cancer. Therefore, this review aimed to evaluate the impact of cancer-related LEL on patient QOL and psychosocial well-being. Specific objectives include:

(1) Summarize and critically evaluate the literature on the impact of cancer-related LEL on patient QOL and psychosocial well-being.
(2) Identify potentially modifiable factors associated with poor QOL and psychosocial well-being in patients with cancer-related LEL.

## 2. Experimental Section

### 2.1. Methods

This systematic review followed the Preferred Reporting Items for Systematic Reviews and Meta-analyses (PRISMA) guidelines [20]. Three electronic databases were searched: PubMed, PROQuest, and Scopus. The search terms and strategy used within PubMed are outlined within Appendix A (Table A1). Terms were categorized into four domains: lymphedema (e.g., “lymphedema”, “lymphoedema”, and “secondary lymphedema”), lower-extremity (e.g., “lower-extremity”, and “lower limb”), cancer-related (“neoplasm”, “cancer-related”, and “oncology”), and psychosocial well-being/QOL (e.g., “quality of life”, “well-being”, and “anxiety”). The search strategy was modified and applied to PROQuest and Scopus with the aid of a professional librarian.

The inclusion criteria included articles: (1) published prior to 1 May 2020; (2) that examined relationships between cancer-related LEL and global QOL; (3) and/or at least one of the following psychosocial domains: psychological distress (e.g., depression, anxiety, or any other mood state), physical, social, family, emotional, or functional well-being, sexual function, body image, or financial implications (primary or secondary measures). The exclusion criteria were articles published in non-English languages and/or those published before 1 January 2000.

### 2.2. Data Extraction

An initial search was conducted by a professional librarian and the search strategy was refined in collaboration with two reviewers (C.B. and K.-A.P.). Following PRISMA guidelines (Table A2), two reviewers (C.B. and K.-A.P.) completed the refined literature search, eligibility screening, data extraction, and performed quality assessments of the articles included. Discrepancies between reviewers were discussed until consensus was met. After removing duplicates, each reviewer screened titles and abstracts using the aforementioned inclusion/exclusion criteria (level 1). Remaining abstracts were then screened as full-text articles (level 2) and data extraction was undertaken using a previously designed form [21]. Data extraction summarized details including study objectives, patient demographics and disease-related characteristics, lymphedema status determination, design and data collection methodology, and psychosocial and/or QOL-related findings. If raw instrument scores were reported, differences in overall and subscale scores were calculated and verified by two authors (C.B. and K.-A.P.). Literature synthesis was performed by reading all the articles several times to become familiar with the results, methods used and methodological limitations. After summarizing the findings, studies with similar and conflicting results were identified and supporting data were extracted from articles. Conclusions from this review were then made.

### 2.3. Quality Assessment

Quality assessments of included articles were undertaken using the National Institutes of Health (NIH) Quality Assessment Tools [22]. These tools include appraisal criteria specific to study design. For instance, observational cohort and cross-sectional studies were rated as “poor”, “fair”, or “good” based upon the following criteria: exposure-related considerations (timeline relative to outcome measurement, frequency of measure, and categorization of exposure levels), methodological validity of exposure and outcome measurements, participation and post-baseline follow-up rates, adjustment for confounding variables, outcome assessor blinding, and explicitness of aims, sample, and study setting.

## 3. Results

**Figure 1:**
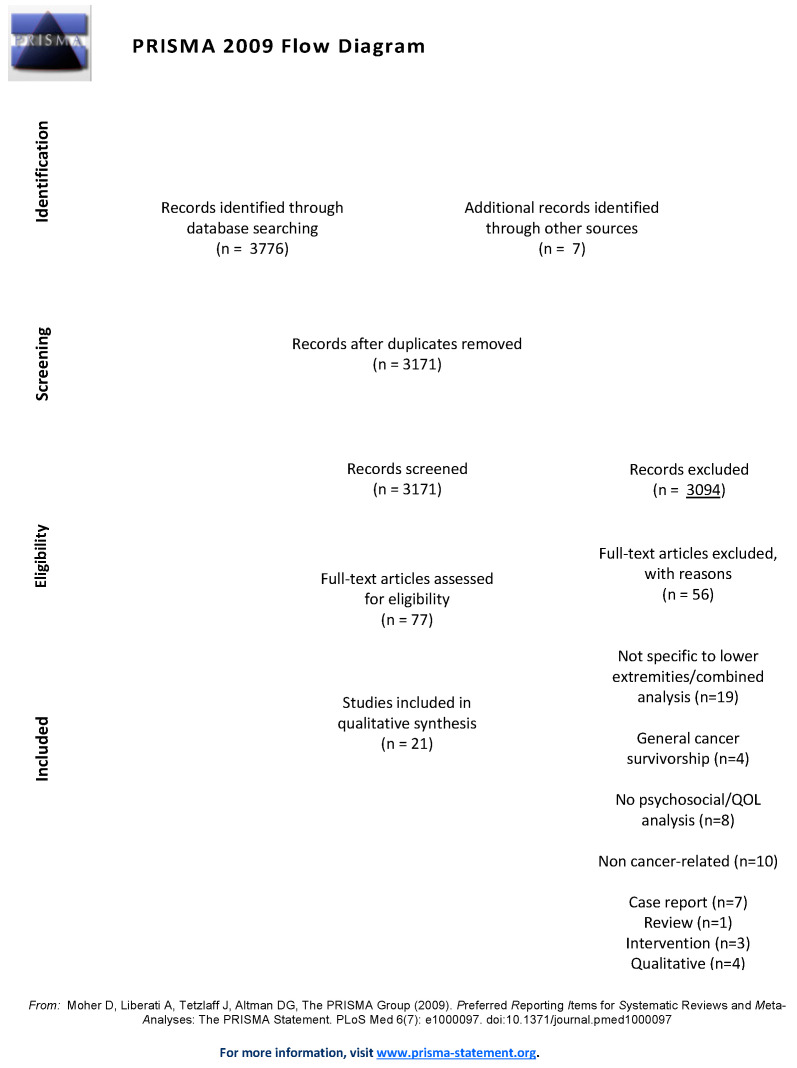
The PRISMA flow diagram demonstrating article screening outcomes and reasons for exclusion [20].

A total of 3171 unique articles were retrieved using the aforementioned search strategy. After screening article titles and abstracts (level 1) for inclusion based upon eligibility criteria, 77 articles remained and were subject to full-text screening. Of these articles, 56 were excluded based upon the following: case reports (*n* = 7), review (*n* = 1), intervention (*n* = 3), solely qualitative analysis (*n* = 4), not specific to lower extremities and/or solely included combined extremity analyses (*n* = 19), addressed general cancer survivorship (*n* = 4), non-cancer-related lymphedema (*n* = 10), or lacked psychosocial/QOL analyses (*n* = 8). A total of 21 articles remained after all screening processes were completed. A PRISMA diagram outlining the literature search and screening outcomes is presented in Figure 1.

### 3.1. Study Characteristics

There was a total of 7500 participants included in the evaluated studies, ranging from 22 to 1243 participants (Table 1) [5,23,24,25,26,27,28,29,30,31,32,33,34,35,36,37,38,39,40,41,42]. The calculated mean age of participants across all studies was 60.7 years and participants were mostly women with a history of gynecological cancer. Studies were conducted in the USA (*n*= 7), Australia (*n* = 6), the Republic of Korea (*n* = 2), Brazil (*n* = 1), Denmark (*n* = 1), Italy (*n* = 1), Japan (*n* = 1), Sweden (*n* = 1) and the United Kingdom (*n* = 1) [5,23,24,25,26,27,28,29,30,31,32,33,34,35,36,37,38,39,40,41,42].

Overall, the studies were primarily cross-sectional (*n* = 6) [38,39,40,41,42,43], prospective cohort studies (*n* = 6) [24,25,26,30,31,32], retrospective cohort studies (*n* = 6) [5,27,28,29,33,35], and case–control studies (*n* = 3) [23,34,36]. Study design was also assessed based upon the specific methodology implemented when investigating LEL in relation to QOL/psychosocial well-being, which in some cases was different than the overall study design. This sub-analysis revealed cross-sectional (*n* = 16) [5,23,24,25,26,27,28,29,34,36,37,38,39,40,41,42], prospective cohort (*n* = 3) [30,31,32], and retrospective cohort (*n* = 2) [33,35] methodologies.

Cancer-related LEL was most often identified through self-reported questionnaires (*n* = 13) [5,19,23,25,27,29,30,32,36,37,38,39,42], of which four reported questionnaire validity and five incorporated a secondary clinical assessment. Limb volume and tissue changes assessed by a health care provider were also used for LEL identification (*n* = 4) [24,30,40,41,42], formal clinical diagnosis (*n* = 2) [32,44], and clinical examination with lymphatic imaging (*n* = 2) [40,42]. Three articles did not report on lymphedema identification criteria [26,27,28]. The majority of studies (*n* = 14) focused on QOL (global or HRQOL) and/or one or more psychosocial domains as the primary study aim [5,24,26,27,28,30,31,33,35,36,37,40,41,42], while the remaining seven studies focused on cancer-related LEL prevalence/incidence as the primary aim, with psychosocial measures as secondary outcomes [23,25,29,32,34,38,39]. In order to assess dimensions of QOL and psychosocial well-being, studies either used one or more of the following measures: twelve studies used the European Organisation for Research and Treatment of Cancer (EORTC) instruments, five used Functional Assessments of Chronic Illness Therapy (FACIT) tools, four used the Hospital Anxiety and Depression Scale (HADS), three used the Medical Outcomes Survey Short Form-12 (MOS SF-12), and two used the Gynecologic Cancer Lymphedema Questionnaire (GCLQ). Other measures of QOL and psychosocial well-being are listed within Appendix A (Table A3).

### 3.2. Quality Assessments

Quality assessments are provided in Table 2 and Table 3, and a summary of key findings is reported below.

#### 3.2.1. Observational Cohort Studies

Five studies used an observational cohort design [30,31,32,33,35]. The study by Cromwell et al. [30] was the only observational cohort study to receive a ‘good’ quality rating. The study was adequately powered (*n* = 277) and used a clinically valid method to identify LEL cases (≥10% volume increase at three months post-baseline), while further identifying and assessing QOL and psychosocial well-being in patients “at risk” for and/or with lower severity LEL using validated outcome measures.

The remaining studies were appraised as “fair” (*n* = 3) [32,33,35] and “poor” (*n* = 1) quality [31]. The work by Franks et al. [31] received a “poor” rating due to several methodological limitations and unmet criteria on the NIH Quality Assessment Tool. LEL identification relied upon self-reported ‘prolonged swelling that fails to resolve on bedrest and/or an indication of cancer within medical records, both of which are not individually sufficient for formal cancer-related lymphedema diagnosis/identification. Furthermore, HRQOL scores for LEL patients with ulcerations were not directly compared to normative values; rather, scores were compared between these patients and individuals with leg ulcerations. The authors deduced that because ulcerations can cause major deficits in HRQOL, lymphedema patients are more likely to experience greater problems.

#### 3.2.2. Cross-Sectional Studies

Sixteen studies used a cross-sectional design, of which twelve were rated as “fair” [23,24,25,26,28,29,34,37,40,41,42]. For example, a study by Gjorup et al. [41] used clinically relevant LEL identification criteria, multiple validated outcome measures, and adjusted for potential confounding variables (e.g., age and sex). Four studies received a rating of “poor” [5,27,38,39]. Across all studies, a major shortcoming was an inability to explore exposure-outcome directionality due to inherent cross-sectional design limitations. Other factors included no reporting of assessor blinding status, lack of lymphedema staging/severity sub-analysis, and single exposure measurements.

### 3.3. Summary of Outcomes

Twenty-one studies evaluated the implications of cancer-related LEL on global QOL and/or one more of the following QOL domains: (i) physical and functional well-being (i.e., fatigue, pain, ADLs); (ii) psycho-emotional well-being; (iii) social, relational and financial well-being.

#### 3.3.1. Global QOL Implications

The majority of studies evaluated the relationship between LEL status and global QOL scores and all found a negative correlation. For instance, in one cross-sectional study by Dunberger et al. [5] (*n* = 616), gynecological cancer survivors with cancer-related LEL were more likely to report lower overall QOL scores than participants without LEL (RR = 1.4). In another prospective, longitudinal study of cervical cancer patients, Ferrandina et al. [26] found that the global health status (EORTC QLQ-C30 and CX24) was associated with early worsening of LEL following cancer treatment, which was common across women treated for early stage and locally advanced cervical cancers [26]. Taken together, data from these studies suggest that LEL correlates negatively with QOL.

#### 3.3.2. Physical and Functional Implications

Many of the studies assessed physical and/or functional well-being in relation to cancer-related LEL status and reported a negative relationship between these factors. In an observational cohort study of 147 women who received treatment for cervical, endometrial, or ovarian cancer, Omichi et al. [33] reported FACT-G scores within the physical domain were significantly lower in patients with LEL compared to those without LEL. Similarly, in a cohort study by Stolldorf et al. [35] (*n* = 178), participants with cancer-related LEL reported higher physical symptom scores (16.1% of participants). These findings were supported by cross-sectional analyses [23,24,25,29,34,36,37,38,39]. It should be noted, however, that two studies failed to show significant results [39,42]. A cross-sectional case–control study by Kim et al. [42] did not report significant differences in EORTC QLQ-C30 (functional and physical symptom domains) scores between 25 women with gynecologic cancer-related LEL and 28 gynecological cancer survivors without LEL, potentially due to a small sample size that resulted in low statistical power. Brown et al. [39] also indicated no significant differences in patient-reported changes to physical activity between women with cancer-related LEL and those without LEL. Nevertheless, this same study reported a significantly increased odds of reporting poor physical function within the LEL group compared to the non-LEL group (OR 5.25) [39]. Overall, findings from these studies suggest that cancer-related LEL status negatively influences the physical and functional domains of patient QOL, but further suggest a need for increased research in order to better under the specific effects of lymphedema on these domains.

#### 3.3.3. Pain and Fatigue

Although pain and fatigue may not be considered direct signs of lymphedema, many of the studies described the incidence or frequency of pain and fatigue, and suggested that they were commonly reported with negative effects on QOL [23,24,29,32,38,41,46]. For instance, pain was one of the most commonly reported physical symptoms in a study of 38 cancer survivors with LEL [38]. Gane et al. [32] reported tingling and weakness of moderate or severe intensity amongst cancer-related LEL patients. The presence of these symptoms was associated with lower QOL (any symptom estimate −13.29; moderate to extreme symptom estimate −11.35) [32].

#### 3.3.4. Activities of Daily Living

Within the context of physical and functional QOL, some studies found that cancer-related LEL was associated with physical limitations and unmet needs, thus interfering with ADLs [34,38,41,47]. For example, in one study by Beesley et al. [34], patients with endometrial cancer-related LEL had a higher odds ratio (OR) for physical or daily living unmet needs (OR 2.21) when compared to endometrial cancer survivors without LEL. Furthermore, 3% of these participants had mild difficulty completing ADLs, whereas 26% had moderate and 5% had severe difficulty, illustrating the varying impact of LEL on daily activities, which is likely associated with varying severity of LEL itself. These findings were echoed within a cross-sectional study by Kusters et al. [37] (*n* = 160), which found that gynecological cancer survivors with self-reported LEL (30%) described significantly more unmet needs relating to physical/daily living, health needs, and patient care after 5–30 months of receiving a cancer diagnosis (SNCS SF-34) [37]. Taken together, data from these studies indicate that the repercussions of LEL on ADLs need to be addressed in an attempt to improve patient physical and functional well-being and subsequently, overall QOL.

#### 3.3.5. Psycho-Emotional Implications

Eighteen studies assessed psychological and emotional well-being in relation to cancer-related LEL status and found a negative relationship [5,24,25,26,27,28,29,30,31,32,33,35,36,37,40,41,42,46]. For instance, Yost et al. [29] reported that women with endometrial cancer-related LEL experienced significantly lower psycho-emotional QOL than survivors without LEL. Omichi et al. [33] demonstrated that these effects were sustained over time, in which gynecological cancer survivors with LEL reported lower overall emotional QOL than those without LEL 3–6 months, and 9–12 months after cancer treatment. Of the eighteen studies, five studies examined LEL-specific psychological sequelae and potential sources of poor psycho-emotional outcomes [5,19,27,37,41]. For instance, Dunberger et al. [5] found that patients with cancer-related LEL were more likely to report fear of cancer recurrence (RR 1.3) than other cancer survivors. Stolldorf et al. [19] reported that among 37 patients with cancer-related LEL, 15.3% of participants experienced increased sadness, 17.2% experienced anger, 10.3% experienced a lack of self-confidence, 15.4% had concerns about their appearance, and 15.5% experienced a loss in body confidence related to their LEL [19]. Furthermore, a cross-sectional analysis by Kusters et al. [37] (*n* = 160) found that gynecological cancer survivors with LEL reported distress, high levels of depressive and anxiety-related symptoms more frequently than patients without LEL (HADS depression score ≥ 8, 29%; HADS anxiety score ≥ 8, 31%). Taken together, these studies suggest that heightened psychological and emotional distress is common among patients with cancer-related LEL and can impact QOL.

#### 3.3.6. Social, Relational and Financial Implications

Ten studies evaluated the impact of cancer-related LEL on the social, relational, and financial dimensions of one’s life and suggested a negative association [5,23,24,28,29,30,34,35,36,42]. For instance, within two studies by Beesley and colleagues [23,34], patients with endometrial cancer-related LEL experienced unmet needs relating to the cost of LEL and management of LEL within the workplace. In addition, both Yost [29] and Rowlands [36] reported patients with LEL had significantly lower social domain scores on the EORTC QLQ- and MOS SF-12 relative to non-LEL comparison groups. With regard to relational QOL outcomes, Dunberger et al. [5] found no difference in perception of sexuality and prevalence of feeling attractive as women between 218 gynecological cancer survivors with LEL and 388 counterparts without LEL. It may be the case that gynecological cancer survivors are highly vulnerable to sexual dysfunction with or without lymphedema. However, experiences of poor body image and sexual dysfunction among patients with LEL were reported in three other studies [28,29,35]. For instance, one study reported that among 37 patients with cancer-related LEL, 11.5% of participants felt misunderstood by their significant other, less sexually attractive (13.8%), lacked interest in sex (21.4%), and unable to take part in sexual activity (18.1%).

### 3.4. Modifiable Factors Associated with Poor QOL in Patients with Cancer-Related LEL

While several studies evaluated the impact of cancer-related LEL on patient QOL and psychosocial well-being, very few provided insight into potentially modifiable factors related to QOL and suggested that a greater number of LEL-related symptoms and higher LEL severity were associated with poorer QOL. For instance, Kim et al. [42] found that high GCLQ-K LEL symptom scores were negatively and strongly associated with overall global health (EORTC QLQ-C30) scores (r = |0.64|) of LEL patients (Cohen’s *d* = 1.26) and more strongly correlated with physical, role, emotional and social function, symptom scales, dyspnea, constipation and financial difficulties EORTC QLQ-C30 domains (range across domains; r = |0.31–0.72|). Gane et al. [32] also reported on the negative influence of lower limb symptom prevalence and severity in relation to patient QOL.

## 4. Discussion

The purpose of this systematic review was to evaluate the impact of cancer-related LEL on patient QOL and to identify potential factors associated with worse QOL among cancer patients with LEL, particularly modifiable factors. The evidence available to date suggests that cancer-related LEL adversely impacts the physical, functional, psycho-emotional, social, relational and financial domains of patients’ QOL. A greater number of LEL symptoms and higher LEL severity may catalyze those negative effects, indicating that proper management of modifiable factors (LEL symptoms and severity) may have a positive impact on patient health and well-being. The findings from studies included in this review are supported by the qualitative literature, showing unique factors underlying the LEL experience may diminish patient QOL [17,44,45,47]. For example, Ryan et al. [17] described patients’ negative experiences with the frustration of symptoms and treatment, diminished self-confidence and poor self-perception, and changes to body image. These findings were further supported by LEL patients’ experiences of isolation, depressive thoughts, and fears of the future, all of which are implicated in patient QOL and psychosocial well-being [17,44].

Five of the twenty-one studies incorporated a temporal analysis (retrospective or prospective cohort design) [30,31,32,33,35], and only one of them received a quality rating of “good”. This study concluded that patients with LEL had significantly lower QOL than those with upper-extremity lymphedema [30], with LEL patients also experiencing declining QOL over the course of 18 weeks after cancer treatment. This effect was not shared by other subtypes of lymphedema [30]. Three of the remaining observational cohort studies were appraised as “fair” [32,33,35] and showed that patients with LEL had significantly worse QOL compared to other cancer survivors without LEL. One of these studies also reported a negative association between LEL symptom prevalence and severity, and multiple QOL domains [32]. Sixteen studies included within this review employed a cross-sectional design [5,23,24,25,26,27,28,29,34,36,37,39,40,41,42]. The majority were rated as “fair”, and supported previous findings from observational cohort studies [30,31,32,33,35]. Overall, while the literature supports a relationship between cancer-related LEL and poor QOL, the quality of evidence is currently insufficient since most studies were cross-sectional, which precludes conclusions regarding directionality or temporality.

### 4.1. Implications for Research and Clinical Practice

This work is one of the first systematic reviews to provide insight into the QOL, unmet needs and psychosocial well-being of cancer survivors with LEL. This review reveals that research in this area is limited, with the majority of studies lacking temporal and directional analyses due to the use of cross-sectional designs. Of note, longitudinal analyses amongst breast cancer-related upper-extremity lymphedema patients demonstrate worsening of QOL and psychosocial functioning as time passes after cancer treatment [48,49]. Considering the unique challenges faced by patients with LEL, who require sufficient function of lower limbs for basic mobility, longitudinal analyses are warranted to examine the QOL implications associated with LEL chronicity and progression.

Although formalized clinical programs are available to aid cancer survivors in addressing common complications of cancer treatment, such as fatigue and distress, few oncology programs provide direct support for cancer-related LEL management, leaving many patients to navigate the complex physical and psychosocial burdens associated with this disease [50,51]. This review demonstrates that LEL patients experience heightened levels of the many long-term common complications of cancer treatment compared to survivors without LEL. These complications are, in part, linked to lymphedema-specific factors, including LEL symptom prevalence and severity. Therefore, this review provides critical information for survivorship program development, emphasizing the profound need for LEL-specific management, clinical guidelines and tools in order to support patients after active cancer treatment and improve their QOL.

This review indicates that evidence is consistent as to whether cancer-related LEL negatively impacts QOL and psychosocial well-being. Studies have assessed QOL and psychosocial well-being at a variety of time points during cancer survivorship, indicating that patients with LEL may require additional assistance to manage their distress effectively across the cancer continuum. In addition, research describing modifiable factors associated with QOL in this population is limited, suggesting a greater number of LEL symptoms and higher LEL severity are associated with poor QOL [32,42]. These studies have focused on medical correlates of QOL, with little attention to the potential influence of psycho-behavioral characteristics. For example, self-efficacy and self-care management are two factors that are modifiable and have been found to moderate the effects of chronic illness on QOL [52,53], yet this relationship has not been examined in patients with cancer-related LEL. These and other potentially modifiable factors need to be assessed and identified to allow for their inclusion in future strategies aimed towards improving QOL and psychosocial well-being within this understudied population.

### 4.2. Limitations

There are several limitations associated with this systematic review. To begin, the heterogeneity of studies in terms of study design and measures prevented pooling the data in a meta-analysis, and therefore, an analysis of risk of publication bias could not be conducted. Furthermore, studies that conducted combined primary lymphedema, secondary non-cancer-related lymphedema and cancer-related LEL analyses were not included. It is possible that these studies presented important information pertaining to QOL and psychosocial well-being of patients living with cancer-related LEL. However, the heterogeneity among participants with and without a history of cancer was deemed inappropriate for inclusion within this review. Furthermore, some studies on cancer-related LEL may have been missed due to the databases searched in this review. Additional literature searches in databases such as CINAHL and PsychInfo may have provided a greater breadth of studies on practical LEL literature and psychological studies.

Limitations also existed within the literature included within this review. Included studies lacked age- and sex-related diversity of study participants. Sixteen of the twenty-one articles included focused on women with LEL related to gynecological cancer treatment [5,23,24,25,26,27,28,29,32,33,35,36,38,39,42,46]. Furthermore, articles that reported on other cancer types, such as melanoma and colorectal cancer, primarily included female participants. This finding not only emphasizes the need for more diversity related to disease characteristics and demographics, but further suggests a potential imbalance in patients seeking medical care and/or reporting lower-extremity swelling and symptoms within the literature. To further augment demographic limitations, the mean age of participants included within this literature review was 60.7 year, representing a relatively older cohort of survivors. Although this is representative of the population of people typically diagnosed with cancer, without younger survivors included, the full spectrum of survivorship experiences is not identified. For example, several studies emphasized the financial burden associated with cancer-related LEL in regards to treatment-related economic strain and patients’ difficulty with symptom management at work. Considering the differences in employment and financial stability that may exist between older and younger adult cancer survivors [54], it is critical that QOL is also assessed within younger LEL populations. To this end, Gjorup et al. [41] reported that younger women with LEL (all cause) had significantly worse social functioning than older women. It is also the case that younger cancer survivors, in general, tend to encounter more psychosocial issues than their older counterparts [55,56]. Therefore, this review, in combination with previous qualitative studies emphasizing the importance of age in LEL symptom management and coping [45], sheds light upon the complex interplay between age, cancer-related LEL and QOL. This relationship should be addressed in future studies in order to strengthen the psychosocial literature within this context.

## 5. Conclusions

Cancer-related lymphedema is a progressive, lifelong complication of active cancer and its associated treatment. Despite its prevalence, cancer-related LEL continues to be poorly recognized amongst the health care community, leaving many patients to navigate complex psychosocial and QOL implications. Although the current literature suggests a negative relationship between cancer-related LEL and QOL/psychosocial well-being, there is a substantial need for rigorous longitudinal studies to examine the directionality and temporality of this effect.

## Figures and Tables

**Table 1:** Psychosocial LEL Study Data Extraction.

Reference	Participants	Definition of Lymphedema	Design	QOL and Psychosocial Well-Being Outcomes	Conclusion	Strengths and Weaknesses
Leitao et al. 2020 [25]	599 endometrial cancer patients (M_age_ = 61)	13-item LEL PRO survey with ≥5 total score indicative of LEL	Observational: Primary Aim: Prospective Cohort Study Analyses of QOL/Psychosocial Well-Being and LEL: Cross-Sectional	EORTC QLQ-C30 overall and global QOL scores significantly lower in LEL group vs. non-LEL group (mean difference 79.0–91.8; 66.8–83.6); significant decreases across all functional and symptom scale domains in LEL group vs. non-LEL group. EORTC QLQ-EN24 functional (mean difference 21.83–30.67) domain scores were significantly lower in LEL patients vs. non-LEL group; and symptom domain scores were significantly higher in LEL group vs. non-LEL group (mean difference 31.6–14.07).	LEL group had worsened QOL and functional well-being than non-LEL group; sexuality, physical function, and psychological well-being included in QOL assessment.	Strengths: Large sample size, LEL identification tool Weaknesses: Recall bias, no LEL staging
Beesley et al. 2007 [34]	1243 women with endometrial cancer (stage I–IV) (M_age_ = 61)	Defined by three categories: no edema, undiagnosed swelling of unknown cause and diagnosed lymphedema; LE defined by series of questions developed to explore symptomology and experience	Observational: Primary Aim: Case–control Study Analyses of QOL/Psychosocial Well-Being and LEL: Cross-Sectional	LEL patients had higher relative odds (unadjusted) of psychological (OR 2.49; 95% CI, 1.49–4.17; *p* < 0.001), physical or daily living (OR, 2.21; 95% CI, 1.37–3.58; *p* = 0.001); 25% of LEL patients reported moderate/high need for help to be more informed about causes, prevention and LE treatment, and to be given information on LEL; 20% reported unmet need for help with pain/discomfort in legs/groin; 18.8% reported unmet need of managing symptoms of LE in workplace.	Cancer-related LEL resulted in changes in appearance, difficulties completing ADLs, and increased unmet needs. Participants reported variable LEL education pre- and post-operatively.	Strengths: Large sample size, addresses multiple issues related to LEL, multiple LEL classifications and sub-analyses Weaknesses: Cross-sectional design, self-report LEL identification
Dunberger et al. 2013 [5]	789 gynecological cancer survivors (M_age_ = 63.9)	Swelling in legs “at least occasionally” within past 6 months vs. not at all	Observational: Primary Aim: Retrospective Cohort Study Analyses of QOL/Psychosocial Well-Being and LEL: Cross-Sectional	Overall QOL significantly lower in LEL group vs. non-LEL group (RR 1.4, 95% CI 1.2–1.6); decreased sleep satisfaction (RR 1.3, 95% CI 1.1–1.5), fear of cancer recurrence (RR 1.3, 95% CI 1.1–1.5), LEL patients more likely to interpret symptoms/signals from body as indicating recurrence (RR 1.4, 95% CI 1.2–1.7); negative impact on several ADLs, 45% of LEL patients avoided exercise, housework (29%), 27% avoided social activities, 20% avoided meeting friends.	LLL after gynecological cancer treatment has negative impact upon QOL, sleep; few patients seek professional help, difficulties with ADLs.	Strengths: Tools specific to population of study, large sample size Weaknesses: Poor definition of LEL, older population of study (limited generalizability), no non-cancer comparator
Yost et al. 2014 [29]	191 women with endometrial cancer (stage I–IV) (M_age_ = 61.8)	Self-reported lymphedema diagnosis after surgery or screen positive on 13-item LE screening questionnaire	Observational: Primary Aim: Retrospective Cohort Study Analyses of QOL/Psychosocial Well-Being and LEL: Cross-Sectional	LEL had greater negative impact on QOL (30-item QOL) than BMI; QLQ-C30 global (mean difference −11.8) physical (mean difference −9.0), role function (mean difference -8.4), emotional (mean difference −12.0), cognitive (mean difference −10.1), and social (mean difference -−13.1) scores lower in LEL patients vs. non-LEL patients (BMI < 30 kg/m^2^); LEL patients reported significantly higher fatigue (mean difference 18.2), pain (mean difference 15.1), and dyspnea (mean difference 9.5). Adverse impacts on QOL were worst with patients with LEL and obesity/high BMI vs. no LEL and obesity/high BMI (mean difference global QOL −22.1); LEL patients also reported worse endometrial cancer-specific domains on 24-item endometrial cancer module.	LEL has negative impact on psychosocial well-being and QOL; adverse events associated with sexual function, body image, urological symptoms reported more by LEL patients than non-LEL group.	Strengths: Tools specific to population of study, large sample size, clinical significance sub-analysis Weaknesses: Poor definition of LEL, homogenous sample of older population self-report measures, deductive interpretation of QOL based on BMI (indirect)
Beesley et al. 2015 [23]	1243 women treated for endometrial cancer (stage I–IV) (M_age_ = 61)	Self-report swelling in legs/feet/groin and/or history of LE diagnosis; EMR analysis of LE-related treatments/appointments <3 years	Observational: Primary Aim: Case–control Study Analyses of QOL/Psychosocial Well-Being and LEL: Cross-Sectional	55% of women with self-reported LEL conveyed a need for help with LEL-specific issues; 29% had at least one moderate- to high-level LEL-specific need that was unmet associated with cost of LEL, pain and discomfort of affected region.	LEL patients have unmet LEL-specific needs, including physical symptoms and financial challenges.	Strengths: Multiple LEL-specific measures (physical, care needs), large sample size Weaknesses: Poor definition of LEL, lacking tool validation
Cromwell et al. 2015 [30]	277 patients with melanoma; 135 male and 142 female; age <50 n = 106 and ≥50 n = 171	≥10% volume increase compared to baseline (3 months post); 5–10% increase indicative of “at risk” for LEL	Observational: Prospective Longitudinal Cohort Study	At-risk LEL patients had higher coping efficacy (13-item coping efficacy with LE instrument) (9–12 months post) than at baseline, and upper-extremity LE patients; mild/moderate-LEL patients improved in coping over 18 months (OR 7.2, 95% CI: 3.5–14.5) and performance of ADLs (OR 6.8, 95% CI: 3.2–14.3). Improvements in coping associated with LE (*p* = 0.08), and higher reported interference with ADLs (OR: 2.5, CI 1.3–5.0). LEL (vs. UEL) had lower FACT-M scores (OR 2.43, 95% CI: 1.34–4.35, *p* < 0.01). LEL patients had a higher score for impact of LEL on ADLs (12-item effects of LE on ADL); high variation in sleep, leisure activities, and choice of clothing associated with LEL; decrease in FACT-M (50-item) scores with time (associated with LEL, did not occur with non LEL).	Overall FACT-M decrease over time associated with LEL group; differences in coping, ADLs, and physical symptomology noted with LEL.	Strengths: Unique measure of coping efficacy, assessed function and psychosocial factors associated with LEL using multiple measures, longitudinal study on coping efficacy Weaknesses: Few patients treated with radiation (high LEL correlation), some quantitative data (coping efficacy) not tabulated; inconsistent reporting of ORs, QOL scores
Kim et al. 2015 [42]	25 gynecological cancer survivors with LLL and 28 gynecological cancer survivors without LLL (stage I–IV) (M_age_ = 50.8)	Physical exam and limb volume measurement by perometry, lymphoscintigraphy, MRI, and CT; DVT excluded via limb sonography/CT venography	Observational: Cross-Sectional	EORTC QLQ-C30 scores across functional (mean difference 80.3–83.1), symptoms (mean difference 15.0–10.57), and additional symptoms (15.0–10.6) domains were not significantly different between LEL group and control; financial difficulty more commonly observed in LEL population (mean difference 16–6.0); global health status deemed poorer in LEL group (mean difference 62.7–71.4, ns *p* = 0.069); higher number of symptoms more strongly correlated with EORTC (R value range |0.09–0.72|) and global health scores (R value |0.64|). GCLQ-K total symptom scores significantly higher in LEL group vs. non-LEL group, also strongly associated with global health status in EORTC QLQ-C30 (mean difference 5.32–1.86). Cohen’s *d = 1.26.*	LEL group did not have lower QOL scores than control group; however, increased financial difficulty associated with LEL was reported. GCLQ-K total symptom score/scores for swelling-general and swelling-limb/heaviness were significantly higher in the LLL group than control.	Strengths: Strong LEL identification, diversity of cancer stage and type Weaknesses: Cross-sectional design, lack of sequential changes in LEL symptoms/related well-being, no associations provided, only reports on patients with clinical diagnosis, no self-reporting
Kusters et al. 2015 [37]	160 gynecological cancer survivors (stage I–IV) (M_age_ = 61)	GCLQ—sensitivity and specificity for LEL for scores of 4 or more symptoms of lymphedema. Self-reported	Observational: Cross-Sectional	LEL patients had a higher mean score (SCNS-SF34) of unmet needs in psychological (*p* = 0.01), physical and daily living (*p* = 0.04), health needs (*p* = 0.02), and patient care needs (*p* = 0.02) relative to non-LEL group. GCLQ: Reported significantly lower physical well-being (*p* = 0.008) and lower functional well-being than non-LEL group. FACT-G: Functional well-being (ns *p* = 0.08) and overall QOL (ns *p* = 0.08); women with LEL needed more services. Higher reporting of distress by women with LEL. Number of participants using or needing each service is provided in Table 2. Depression (HADS, 29%) or anxiety (HADs, 31%) were more commonly reported by LEL group vs. non-LEL group (19% and 21%, respectively).	Psychological distress and unmet needs higher in women with LEL vs. non-LEL group; overall functional and QOL decrease in LEL group.	Strengths: Strong LEL identification, diversity of cancer stage and type, multiple validated tools, explored unique aspect of unmet needs and services used Weaknesses: Cross-sectional design, lack of sequential changes in LEL symptoms/related well-being, instrument scores not provided
Rowlands et al. 2014 [36]	639 women 3–5 year post-endometrial cancer; 68 with LLL, 177 with LLS, and 394 without LLL/LLS (M_age_ = 65.3)	Self-report questionnaire regarding swelling in legs/feet/groin and confirmed history of LE diagnosis by physician	Observational: Primary Aim: Case–control Study Analyses of QOL/Psychosocial Well-Being and LEL: Cross-Sectional	Women with LEL scored significantly lower on QOL scale (MOS SF-12); physical QOL mean difference (41.8 LEL vs. 45.1 non LEL, ns *p* = 0.07) and mental QOL mean difference (49.6–50.6, ns *p* = 1.0) no different in LEL than non-LEL group (Cohen’s *d* = 0.34 and 0.22, respectively). On three of eight MOS SF-12 subscales (physical function 41.1 vs. 44.8, *p* = 0.03, physical role limitations 42.6 vs. 46.2, *p* = 0.03, social function 46.6 vs. 49.6, *p* = 0.04), LEL group scored lower QOL than non-LEL group (Cohen’s *d* = 0.20, 0.39 and 0.45, respectively).	LEL associated with decreased physical functioning and social functioning, increased physical role limitation, ADLs/social activities limited. Summary physical and mental QOL no different between LEL group and non-LEL group.	Strengths: Well-validated QOL instrument, sample size, differentiation between LLS and LEL for analyses, clinical and self-report LEL identification, effect sizes provided Weaknesses: Restricted timeline post-op, cross-sectional design (directionality cannot be obtained)
Stolldorf et al. 2016 [35]	178 patients with secondary (cancer related *n* = 37, non-cancer related *n* = 45) and primary (*n* = 96) LEL (M_age_ = not reported)	Indication of LEL from health care provider, self-report by patient	Observational: Retrospective Cohort Study; Mixed Methods	LSIDS-L scores of cancer-related LEL patients were as follows: Symptom prevalence: sadness (15.3%), anger (17.2%), fatigue (15.5%), difficulty sleeping (15.9%), increased appetite (17%), physical activity (16.1%). Psychological symptom prevalence: lack of self-confidence (10.3%), concerns about appearance (15.4%), loss of body confidence (15.5%). Social and sexual symptom prevalence: misunderstood by SO (11.5), less sexually attractive (13.8%), lack of interest in sex (21.4%), cannot do hobbies or leisure activities (16.4%), social activities (13.3%), sexual activity (18.1%). Insurance-related symptoms: insurance frustration (14.3%).	LEL of all causes impairs QOL and psychosocial well-being. Cancer-related LEL-specific concerns included fear of cancer recurrence, insurance-related concerns, social concerns, financial resources, inability to work, changes to sleep.	Strengths: Large sample size, clear definition of cancer-related and non-cancer-related LEL, LEL identification criteriaWeaknesses: Limited recruitment strategies, self-report clinically confirmed cases
Kim et al. 2010 [27]	828 cervical cancer survivors and 500 controls (M_age_ = 50.4)	Not Provided	Observational: Primary Aim: Retrospective Cohort Study Analyses of QOL/Psychosocial Well-Being and LEL: Cross-Sectional	Lymphedema was significantly associated with increased HADS-anxiety scores within the cervical cancer survivor cohort (univariate analysis). Quantitative data not provided.	LEL associated with increased anxiety after treatment for cervical cancer.	Strengths: Multiple associations between anxiety, depression, and patient characteristics explored, defined patient populationWeakness: Univariate analysis data not provided, no description of LEL identification criteria; single tumour group (limited generalizability)
Ferrandina et al. 2012 [26]	227 cervical cancer patients (M_age_ = 49.3)	N.P.	Observational: Primary Aim: Prospective Longitudinal Cohort Study Analyses of QOL/Psychosocial Well-Being and LEL: Cross-Sectional	EORTC QLQ-C30 and CX24 scores (LEL) revealed early worsening of LEL post-treatment for cervical cancer survivors (difference of 14.6% mean values 12 months compared to baseline in ECC group *p* = 0.001, 28.3% difference in mean values compared to baseline in LACC group, *p* = 0.0001).	LEL considered most disabling treatment-related sequelae explored within study; Patients reported decreased quality of life scores, and several sociodemographic features were associated with the presence of lymphedema.	Strengths: Longitudinal observation; validated and well-established psychometric tools used Weakness: Single tumour group (limited generalizability), lacking description of LEL identification tools
de Melo Ferreira et al. 2012 [24]	28 women with vulvar cancer and 28 healthy, age-matched women (M_age_ = 66.9)	Miller’s Clinical Evaluation; clinical traits included volume, inspection, palpation, changes with limb elevation, and function/joint mobility of limbs	Observational: Primary Aim: Prospective Cohort Study Analyses of QOL/Psychosocial Well-Being and LEL: Cross-Sectional	Occurrence and severity of LLL was significantly greater in cancer survivor group vs. control group (67.9% vs. 10.7%, *p* < 0.001). Severity of LEL was correlated with lower EORT QLQ-C30 scores (total = 0.73, physical = 0.75, cognitive = 0.45, emotional = 0.49, social = 0.57, fatigue= 0.74, pain = 0.78, sleep = 0.74, and financial = 0.70 domains, *p* < 0.05); No association reported between LLL development and sexual and/or urinary dysfunction.	LEL has negative effect on QOL across several psychosocial domains; however, LEL not associated with sexual and/or urinary dysfunction after treatment for vulvar cancer.	Strengths: Multiple outcomes relating to psychosocial well-being and QOL analyzed, quantitative tool- and clinical-based LEL identification Weaknesses: Single tool used to assess QOL
Franks et al. 2006 [31]	189 patients with swollen lower limb (M_age_ = 76.6)	Lower limb(s) swelling present >3 months, fails to completely resolve on bed rest; if <3 months, must have a precipitating factor suggestive of LEL	Observational; Prospective Cohort Study	Largest effect sizes in cancer-related LEL cohort were observed with affective pain (−0.41) and role physical (0.40), but were not statistically significant. For all cause LEL, poor health scores/floor effect scores (SF-36) were reasonable, except physical and emotional; effects were deemed high (0.33 and 0.53).	LEL significantly affects QOL, whether related to cancer or other causes.	Strengths: Large sample size, multiple well-validated tools and cross-comparisons drawn Weakness: Comparisons drawn to venous ulceration population SF-36 scores and McGill short form pain scores post-24 weeks treatment. Generalized tools used to assess HRQOL
Cheville et al. 2010 [40]	236 patients; 28 with cancer-related LEL (M_age_ = 55.6)	Clinical specialist assessment. Factors included Common Toxicity Criteria v.3.0 for limb and truncal LEL (grade 1–4); confirmed by LE certified therapists.	Observational: Cross-Sectional	Adjusted utility scores for time trade-off exercise (TTO) 0.82 and EQ-5D (0.80), poorest amongst cancer-related LEL group, compared to all other LE location and LE cause subgroups. Mean utility score of all cause and location LE in study was 0.85, and EQ-5D was 0.76.	LEL reduces health utilities; lowest adjusted utility scores amongst cancer-related LEL patients.	Strengths: Well-validated quantitative metrics Weaknesses: Subgroup sample size insufficient for EQ-5D utility estimates based upon 95% CI and sample SDs; sampling bias of patients receiving treatment in clinic
Brown et al. 2013 [38]	107 cancer survivors; 35.4% reported ≥1 symptom associated with LEL (M_age_ = 69.3)	Health care provider clinical assessment or self-reported LEL-associated symptoms	Observational: Cross-Sectional	Common symptoms associated with LLL reported by participants included difficulty walking (*n* = 37; 100%), achiness (*n* = 32; 86%), puffiness (*n* = 28; 76%), and pain (*n* = 27; 73%).	LEL affects multiple domains of physical and functional well-being in cancer survivors.	Strengths: Explored multiple domains of psychosocial well-being Weaknesses: No specific QOL or validated psychosocial metrics used; relatively small sample size of LEL subgroup
Brown et al. 2014 [43]	225 women with history of uterine cancer (stage I–IV); 36% of participants had LEL (M_age_ = 63.6)	GCLQ score of ≥ 5, domains include heaviness, general swelling, limb-related swelling, infection, aching, numbness, and physical function	Observational: Cross-Sectional	Odds of reporting poor physical function increased with LEL presence (*p* < 0.0001): OR of 5.25 (95% CI: 2.41–11.41). Differences in physical activity reporting and walking were insignificant between women with and without LEL.	LEL associated with poor self-reported physical functioning. Associations between walking/physical activity and poor physical function not influenced by LEL.	Strengths: Large sample size, multiple associations explored, well-validated tools Weaknesses: Generic LEL tool, cross-sectional prevents directionality analysis
Gane et al. 2018 [32]	22 women newly diagnosed with vulvar (*n* = 20) and vaginal (*n* = 2) cancer (M_age_ = 57)	Self-reported swelling in the leg, vulva, pelvis, or lower abdomen. Clinical diagnosis: medical record review for LEL diagnosis and/or treatment	Observational: Prospective Longitudinal Cohort Study	Measures: FACT-G, measurement of lower limb symptoms associated with lymphedema (rates the severity). Presence of LEL symptoms (even mild) was associated with reduced QOL, and participants with LEL were more likely to report swelling than non-LEL group. Majority of women reported multiple lower limb symptoms such as pain, tingling, or weakness of moderate or severe intensity throughout the 2 year observation period, and the presence of these symptoms was associated with lower QOL (any symptom estimate −13.29; 95% CI, −19.30 to −7.27; *p* < 0.001; moderate to extreme symptom estimate −11.35; 95% CI, −17.30 to −5.41; *p* < 0.001).	Women with vulvar/vaginal cancer experienced high burden of subjective swelling. LEL symptoms associated with lower QOL.	Strengths: Highlights a small/unique subset, clear definition/measure of LEL, validated questionnaire setWeaknesses: Small sample size, could not conduct competing risk analyses, FACT-G scores not provided, questionnaires used to measure LEL symptoms not validated
Farrell R, Gebski V, Hacker NF, 2014 [28]	63 females with vulvar cancer (M_age_ = 63)	Not Provided	Observational; Primary Aim: Retrospective Cohort Study Analyses of QOL/Psychosocial Well-Being and LEL: Cross-Sectional	Measures: Utility-Based Questionnaire-Cancer, a cancer-specific validated QOL instrument. Symptoms were shown in respect to preferred treatment. Average current health state was rated on a visual analogue scale (74%) of full health. Non-LEL group rated current health 80% vs. LEL group at 72%.	Worse scores for QOL in domains of social activity as well as physical and sexual function; quantitative data not provided (forest plot only).	Strengths: large sample, long follow up, validated questionnaire set, LLL only Weaknesses: Includes preferences for treatments preference for sentinel node biopsy or complete lymphadenectomy.
Gjorup et al. 2017 [41]	443 patients treated for cutaneous melanoma; 86 participants had LEL and 23 had upper-extremity LE (M_age_ = 58)	Detailed history, patient symptoms, physical exam LEL staging via International Society of Lymphology	Observational: Cross-Sectional	Measures: (EORTC QLQ-C30), (EORTC QLQ-BR23), FACT-G (social/family well-being subscale, SWB), HADS. Only mean scores are shown for the following subscales: role/social functions, and fatigue/pain and financial.	Those with LEL scored lower on HRQOL, (no significant difference between ULL and LEL). Age/sex in the associations between lymphoedema and HRQOL: younger women with lymphedema had worse social functioning and women had significantly more.	Strengths: Age and sex were included in the analysis Weaknesses: LEL was only a small subsample, no LEL identification tool
Omichi et al. 2017 [33]	75 patients who had received treatment for gynecological cancers (cervical, endometrial, and ovarian) (M_age_ = 56.5)	Medical record LEL staging via International Society of Lymphology	Observational: Retrospective Cohort Study	Measures: FACT-G. LEL patients had poorer QOL scores in physical (P_1_ = 0.026) and emotional (P_2_ = 0.020) domains. Patients affected by two adverse treatment effects had poorer QOL than those with one or no adverse effects in the physical domain (post-treatment1: *p* = 0.049, *p* = 0.001; post-treatment2: *p* = 0.002, *p* = 0.028) and poorer QOL compared with those with no adverse effect in the domain of EWB at post-treatment1 (*p* = 0.017). CC patients (*n* = 35), with LEL had significantly lower physical QOL than non-LEL patients at pretreatment (*p* = 0.019) and post-treatment1 (*p* = 0.010) and in EWB group at pretreatment, post-treatment1 and post-treatment2 (*p* = 0.016, *p* = 0.007, and *p* = 0.016, respectively).	Participants with LEL had significantly poorer QOL.	Strengths: validated QOL tool (FACT-G), longitudinal analysis (pre-/post-treatment) Weaknesses: Medical record identification of LE (no self-report)

**Table 2:** NIH Quality Assessment for Observational Cohort and Cross-Sectional Studies I.

Criteria	Leitao et al. 2020 [25]	Beesley et al. 2007 [34]	Dunberger et al. 2013 [5]	Yost et al. 2014 [29]	Cromwell et al. 2015 [30]	Kim et al. 2015 [42]	Kusters et al. 2015 [37]	Rowlands et al. 2014 [36]	Stolldorf et al. 2016 [35]
Study objectives stated	✓	✓	✓	✓	✓	✓	✓	✓	✓
Study population defined	✓	✓	✓	✓	✓	✓	✓	✓	✓
Eligible participation rate at least 50%?	X	✓	✓	✓	✓	✓	✓	✓	✓
Participant selection and inclusion/exclusion criteria uniformity	✓	✓	✓	✓	✓	✓	✓	✓	✓
Sample size sufficient and/or described	✓	✓	X	X	X	X	X	✓	X
Exposure measured prior to outcome	X	X	X	X	✓	X	X	X	✓
Sufficient time frame for association between exposure and outcome	X	X	X	X	✓	X	X	X	C.D.
Inclusion exposure level	X	✓	X	X	✓	X	X	✓	X
Exposure measures valid and reliable	✓	✓	X	✓	✓	✓	✓	X	✓
Multiple exposure measurements	X	X	X	X	✓	X	X	X	X
Outcome measures valid and reliable	✓	X	X	X	✓	✓	✓	✓	✓
Outcome assessor(s) blinded	C.D.	C.D.	C.D.	C.D.	C.D.	C.D.	C.D.	X	C.D.
Loss to follow up post-baseline 20% or less	N.A.	N.A.	N.A.	N.A.	✓	N.A.	N.A.	N.A.	C.D.
Confounders measured and adjusted statistically between exposure and outcome	✓	✓	✓	✓	✓	X	X	✓	X
Overall rating	Fair	Fair	Poor	Fair	Good	Fair	Fair	Fair	Fair

C.D.—Cannot Determine. N.A.—Not Applicable.

**Table 3:** NIH Quality Assessment for Observational Cohort and Cross-Sectional Studies II.

Criteria	Ferrandina et al. 2011 [26]	De Melo Ferreira et al. 2012 [24]	Franks et al. 2006 [31]	Cheville et al. 2010 [40]	Brown et al. 2013 [38]	Brown et al. 2014 [45]	Beesley et al. 2015 [23]	Kim et al. 2010 [27]	Farrell et al. 2014 [28]	Gane et al. 2018 [32]	Gjorup et al. 2017 [41]	Omichi et al. 2017 [33]
Study objectives stated	✓	✓	✓	✓	✓	✓	✓	✓	✓	✓	✓	✓
Study population defined	✓	✓	✓	✓	✓	✓	✓	✓	✓	✓	✓	✓
Eligible participation rate at least 50%?	✓	C.D.	✓	✓	✓	X	✓	X	✓	C.D.	✓	✓
Participant selection and inclusion/exclusion criteria uniformity	✓	✓	X	✓	✓	✓	✓	X	X	✓	✓	✓
Sample size sufficient and/or described	X	✓	✓	✓	X	X	X	X	X	✓	✓	X
Exposure measured prior to outcome	X	X	✓	X	X	X	✓	X	X	✓	X	X
Sufficient time frame for association between exposure and outcome	X	X	✓	X	X	X	C.D.	N.A.	X	✓	X	✓
Inclusion exposure level	✓	✓	X	✓	✓	X	X	X	✓	✓	✓	✓
Exposure measures valid and reliable	X	✓	X	✓	✓	✓	✓	X	✓	✓	✓	✓
Multiple exposure measurements	✓	X	✓	X	X	X	X	X	X	✓	X	✓
Outcome measures valid and reliable	✓	✓	✓	✓	X	✓	✓	✓	✓	✓	✓	✓
Outcome assessor(s) blinded	C.D.	C.D.	X	X	X	X	X	X	C.D.	C.D.	C.D.	C.D.
Loss to follow up post-baseline 20% or less	✓	N.A	X	N.A.	N.A	N.A.	X	N.A.	N.A	X	N.A.	C.D.
Confounders measured and adjusted statistically between exposure and outcome	X	X	✓	✓	X	✓	✓	X	X	X	✓	X
Overall rating	Fair	Fair	Poor	Fair	Poor	Poor	Fair	Poor	Fair	Fair	Fair	Fair

C.D.—Cannot Determine. N.A.—Not Applicable.

## Appendix A

**Table A1:** Constructed search strategy for PubMed, which was adapted for other databases used.

Concept	Search Strategy
Lymphedema	(“lymphedema”[MeSH terms] OR “lymphedema”[Title/Abstract] OR “lymphoedema”[Title/Abstract] OR “chronic edema”[Title/Abstract] OR “chronic oedema”[Title/Abstract] OR “secondary lymphedema”[Title/Abstract])
Lower-Extremity	AND (“lower-extremity”[Title/Abstract] OR “lower extremity”[MeSH terms] OR “lower extremity”[Title/Abstract] OR “leg”[MeSH terms] OR “leg”[Title/Abstract] OR “lower-limb” [Title/Abstract] OR “lower body”[Title/Abstract])
Cancer-Related [“cancer”]	AND (“neoplasms”[MeSH terms] OR “neoplasms”[Title/Abstract] OR “sarcoma”[Title/Abstract] OR “carcinoma”[MeSH terms] OR “carcinoma”[Title/Abstract] OR “gynaecology”[MeSH terms] OR “gynaecolog*”[Title/Abstract] OR “gynecology”[MeSH terms] OR “gynecolog*” [Title/Abstract] OR “cervical”[Title/Abstract] OR “melanoma”[Title/Abstract] OR “prostate”[Title/Abstract] OR “metastatic”[Title/Abstract] OR “uterine”[Title/Abstract] OR “myometrial”[Title/Abstract] OR “vulvar”[Title/Abstract] OR “tumour”[Title/Abstract] OR “tumor”[Title/Abstract] OR “survivorship”[MeSH terms] OR “survivorship”[Title/Abstract] OR “survivors”[MeSH terms] OR “survivors”[Title/Abstract] OR “oncology”[Title/Abstract] OR “cancer-related”[Title/Abstract])
Psychosocial Well-Being	AND (“QOL” [Title/Abstract] OR “quality of life” [Mesh terms] OR “quality of life “[Abstract/Title] OR “well-being” [Mesh terms] OR “well-being” [Title/Abstract] OR “health” [Mesh terms] OR “activities of daily living” [MeSH terms] OR “activities of daily living” [Title/Abstract] OR “standard of living” [MeSH terms] OR “standard of living “Title/Abstract] OR “living conditions” [MeSH terms] OR “social conditions” [MeSH terms] OR “social conditions [Title/Abstract] OR “psychosocial” [Title/Abstract] OR “psychosocial support systems” [MeSH terms] OR “psychosocial support systems” [Titles/Abstracts] OR “psycholog*” [MeSH terms] OR “psycholog*” [Abstract/Title] OR “psycho-oncology” [Title/Abstract] OR “psycho-oncology” [MeSH terms] OR “survivorship” [MeSH terms] OR “survivorship” [Title/Abstract] OR “self-management” [MeSH terms] OR “self-management [Title/Abstract] OR “psychosocial well-being” [Title/Abstract] OR “stress” [Title/Abstract], “psychological” [MeSH Terms] OR “psychological” [Title/Abstract] OR “psychology, social” [MeSH] OR “rejection, psychology” [MeSH terms] OR “psychological adaptation” [Title/Abstract] OR “psychology, behavioural” [Title/Abstract] OR “social psychology” [Title/Abstract] OR “sexual dysfunction” [MeSH terms] OR “sexual dysfunction” [Title/Abstract] OR “psychological distress” [MeSH] OR “psychological distress” [Title/Abstract] OR “emotional adjustment” [MeSH terms] OR “emotional adjustment” [Title/Abstract] OR “isolation” [Title/Abstract] OR “postoperative care” [Title/Abstract] OR “mental health” OR “famil*” [Title/Abstract] OR “sexual health” [MeSH terms] OR “sexual health” [Title/Abstract] OR “rehabilitation” [MeSH terms] OR “rehabilitation” [Title/Abstract] OR “emotion*” [Title/Abstract] OR “emotions” [MeSH terms] OR “emotional adjustment” [Title/Abstract] OR “depression” [Title/Abstract] OR “holistic nursing” [MeSH terms] OR “anxiety” [Title/Abstract] OR “social support” [MeSH terms] OR “social support” [Title/Abstract] OR “anger” [Title/Abstract] OR “social” [Title/Abstract] OR “social stigma” [Title/Abstract] OR “social factor” [MeSH terms] OR “social factor” [Title/Abstract] OR “famil*” [Title/Abstract] OR “family” [MeSH terms] OR “family” [Title/Abstract] OR “family health” [MeSH terms] OR “distress” [Title/Abstract] OR “depression” [MeSH terms] OR “depression” [Title/Abstract] OR “patient health questionnaire” [MeSH terms] OR “patient health questionnaire” [Title/Abstract] OR “isolation” [Title/Abstract] OR “fear of cancer recurrence” [Title/Abstract] OR “fear of*” [Title/Abstract] OR “fatigue” [Title/Abstract] OR “hopelessness” [Title/Abstract] OR “immobility” [Title/Abstract] OR “financial” [Title/Abstract] OR “financial support” [Title/Abstract] OR “psychosocial factor*” [Abstract/Title] OR “physical symptom” [Abstract/Title] OR “LYMQOL” [Title/Abstract] OR “functional assessment” [Title/Abstract] OR “appearance” [Title/Abstract] OR “identity” [Title/Abstract])

**Table A2:** The PRISMA checklist outlining the approach implemented within this systematic review (22).

Section/Topic	#	Checklist Item	Reported on Page #
**TITLE**			
Title	1	Identify the report as a systematic review, meta-analysis, or both.	1
**ABSTRACT**			
Structured summary	2	Provide a structured summary including, as applicable: background; objectives; data sources; study eligibility criteria, participants, and interventions; study appraisal and synthesis methods; results; limitations; conclusions and implications of key findings; systematic review registration number.	1
**INTRODUCTION**			
Rationale	3	Describe the rationale for the review in the context of what is already known.	2
Objectives	4	Provide an explicit statement of questions being addressed with reference to participants, interventions, comparisons, outcomes, and study design (PICOS).	2
**METHODS**			
Protocol and registration	5	Indicate if a review protocol exists, if and where it can be accessed (e.g., Web address), and, if available, provide registration information including registration number.	n/a
Eligibility criteria	6	Specify study characteristics (e.g., PICOS, length of follow-up) and report characteristics (e.g., years considered, language, publication status) used as criteria for eligibility, giving rationale.	3
Information sources	7	Describe all information sources (e.g., databases with dates of coverage, contact with study authors to identify additional studies) in the search and date last searched.	3
Search	8	Present full electronic search strategy for at least one database, including any limits used, such that it could be repeated.	3, 28
Study selection	9	State the process for selecting studies (i.e., screening, eligibility, included in systematic review, and, if applicable, included in the meta-analysis).	3
Data collection process	10	Describe method of data extraction from reports (e.g., piloted forms, independently, in duplicate) and any processes for obtaining and confirming data from investigators.	3
Data items	11	List and define all variables for which data were sought (e.g., PICOS, funding sources) and any assumptions and simplifications made.	3
Risk of bias in individual studies	12	Describe methods used for assessing risk of bias of individual studies (including specification of whether this was done at the study or outcome level), and how this information is to be used in any data synthesis.	3
Summary measures	13	State the principal summary measures (e.g., risk ratio, difference in means).	3
Synthesis of results	14	Describe the methods of handling data and combining results of studies, if done, including measures of consistency (e.g., I^2^) for each meta-analysis.	3
Risk of bias across studies	15	Specify any assessment of risk of bias that may affect the cumulative evidence (e.g., publication bias, selective reporting within studies).	3
Additional analyses	16	Describe methods of additional analyses (e.g., sensitivity or subgroup analyses, meta-regression), if done, indicating which were pre-specified.	3
**RESULTS**			
Study selection	17	Give numbers of studies screened, assessed for eligibility, and included in the review, with reasons for exclusions at each stage, ideally with a flow diagram.	3,4
Study characteristics	18	For each study, present characteristics for which data were extracted (e.g., study size, PICOS, follow-up period) and provide the citations.	4–19
Risk of bias within studies	19	Present data on risk of bias of each study and, if available, any outcome level assessment (see item 12).	20–23
Results of individual studies	20	For all outcomes considered (benefits or harms), present, for each study: (a) simple summary data for each intervention group (b) effect estimates and confidence intervals, ideally with a forest plot.	23–25
Synthesis of results	21	Present results of each meta-analysis done, including confidence intervals and measures of consistency.	23–25
Risk of bias across studies	22	Present results of any assessment of risk of bias across studies (see Item 15).	20–23
Additional analysis	23	Give results of additional analyses, if done (e.g., sensitivity or subgroup analyses, meta-regression [see Item 16]).	n/a
**DISCUSSION**			
Summary of evidence	24	Summarize the main findings including the strength of evidence for each main outcome; consider their relevance to key groups (e.g., healthcare providers, users, and policy makers).	25
Limitations	25	Discuss limitations at study and outcome level (e.g., risk of bias), and at review-level (e.g., incomplete retrieval of identified research, reporting bias).	25
Conclusions	26	Provide a general interpretation of the results in the context of other evidence, and implications for future research.	26 + 27
**FUNDING**			
Funding	27	Describe sources of funding for the systematic review and other support (e.g., supply of data); role of funders for the systematic review.	27

**Table A3:** All measures used to assess psychosocial well-being and/or QOL within the reviewed literature.

Instrument Category	Instrument Name
European Organisation for Research and Treatment of Cancer (EORTC)	EORTC QLQ-C30, EORTC EN-24
Functional Assessments of Cancer Therapy (FACIT)	FACT-G, FACT-B (modified for LEL), FACT-M
Hospital Anxiety and Depression Scale	HADS-A, HADS-D
Medical Outcomes Survey Short Form-12	MOS SF-12
Gynecologic Cancer Lymphedema Questionnaire	GCLQ
Other	Coping Efficacy questionnaire, Utility-Based Questionnaire-Cancer, Time Trade-Off (TTO) survey, European QOL tool (EurQOL), McGill QOL questionnaire (MQOL), Paffenbarger Physical Activity Questionnaire (PPAQ), Lymphedema Symptom Intensity and Distress Survey (LSIDS-L), Activities of Daily Living questionnaire (ADLs), the Female Sexual Function Index (FSFI), and the Supportive Care Needs Survey (SCNS SF-34)
